# Deceleration and rebound of hemagglutinin divergence in influenza B/Victoria across COVID-19 NPI phases (2016–2024)

**DOI:** 10.3389/fmicb.2026.1791792

**Published:** 2026-02-25

**Authors:** Yue Zhang, Bin Fu, Cuilian Yu, Sai Liu, Yongan Wang, Zhao Wang, Kezhou Wang

**Affiliations:** 1 School of Laboratory Animal & Shandong Laboratory Animal Center, Shandong First Medical University & Shandong Academy of Medical Sciences, Jinan, China; 2 Swine Disease R&D Center, Shandong SINDER Technology Co., Ltd., Qingdao, China

**Keywords:** COVID-19, hemagglutinin, influenza virus, phylogenetics, virus evolution

## Abstract

The COVID-19 pandemic reshaped global viral transmission and created a natural experiment to test how non-pharmaceutical interventions (NPIs) influence the evolution of co-circulating pathogens. We investigated the evolutionary dynamics of the influenza B/Victoria lineage across China, the United States, and Australia—three countries that implemented distinct COVID-19 policy strategies. Using hemagglutinin (HA), neuraminidase (NA), and nucleoprotein (NP) sequences from GISAID (2016–2024), we performed temporal phylogenetic analyses and estimated rate of genetic distance change. Although the duration and strictness of NPIs varied among the three countries, our analysis revealed a transnational common pattern: the evolution of HA showed a marked slowdown during the NPI period. During the post-relaxation period, the population genetic distance of HA in the three countries showed a highly significant increase compared to the pre-pandemic period (comparison between post-relaxation vs. pre-pandemic period, *p* < 0.001). The magnitude and trajectory of these shifts differed by country, broadly aligning with policy timing and intensity. These findings highlight the sensitivity of influenza evolution to population-level interventions and underscore the value of integrating policy metrics into influenza surveillance and pandemic preparedness.

## Introduction

Seasonal influenza remains a major global health challenge, yet the factors governing its evolution in human populations are difficult to disentangle. The COVID-19 pandemic created a unique natural experiment. The widespread implementation of non-pharmaceutical interventions (NPIs)—such as masking, travel restrictions, and school closures—abruptly reconfigured transmission networks for respiratory viruses (Federici et al., 2018; GBD 2017 Influenza Collaborators, 2019; Madhusudanan et al., 2023). This perturbation offers a rare opportunity to examine how shifts in transmission intensity and host immunity shape viral evolution, particularly for influenza B virus (IBV), a significant contributor to seasonal burden and vaccine mismatch risk (Liu et al., 2022; Smith et al., 2004). With the reported functional disappearance of the Yamagata lineage, understanding the evolutionary drivers of the dominant Victoria lineage (IBV-V) has become increasingly urgent (Marchi et al., 2024).

Population-genetic theory predicts that sharp reductions in incidence and effective population size slow adaptive evolution (Coffin and Swanstrom, 2013; Lynch, 2020). For influenza, such effects should be most pronounced in hemagglutinin (HA)—the primary target of host immunity—relative to the more conserved neuraminidase (NA) and nucleoprotein (NP) (Zhao et al., 2024). Consequently, stringent NPIs were expected to decelerate HA evolution, with subsequent relaxation permitting re-acceleration. The magnitude and timing of these shifts, however, likely varied across countries with divergent policies, vaccination histories, and demographics (Aruffo et al., 2022).

National responses to COVID-19 differed markedly. China enforced sustained, centralized containment; the United States adopted a decentralized and temporally heterogeneous strategy, characterized by stringent measures in spring 2020, followed by partial and localized measures in late 2020–early 2021, with most state-level interventions lifted by mid-to-late 2021; and Australia combined strict border controls with phased domestic relaxation (Dergiades et al., 2022; Hale et al., 2021; Wei et al., 2022). These policy disparities-contextually comparable via indices such as the Oxford COVID-19 Government Response Tracker (OxCGRT) - provide a powerful comparative framework (Drummond and Hasnine, 2023; Leiva et al., 2023). While studies have reported suppressed influenza activity and altered phylogenetic patterns during the pandemic (Gupta et al., 2022; Hammond et al., 2021; Olsen et al., 2020), a systematic, cross-national analysis linking policy intensity to evolutionary metrics of IBV-V remains limited.

Here, we test the hypothesis that COVID-19 NPIs were associated with a detectable deceleration in IBV-V HA evolution, followed by re-acceleration upon relaxation, and that the magnitude of these shifts correlates with national policy stringency. We analyzed curated IBV-V HA, NA, and NP sequences from GISAID (2016–2024) for China, the United States, and Australia, stratifying pre-pandemic, intervention, and post-relaxation periods (Bernasconi et al., 2021; Shu and McCauley, 2017). To mitigate sampling bias, we applied stringent quality controls and sensitivity analyses (including year- and country-stratified random sampling) (Lindegren et al., 2012). We then estimated genetic divergence and rate of genetic distance change using standard phylogenetic workflows (e.g., multiple-sequence alignment, model selection, time-scaled phylogenies/root-to-tip regression) and assessed associations with policy intensity metrics (Abraham et al., 2018; Takeuchi and Kawashima, 2023).

Our study makes three contributions. First, by focusing on a single IBV lineage across multiple countries, we isolate lineage-specific evolutionary signals from general suppression effects. Second, by comparing HA to NA and NP, we test whether observed shifts align with theoretical predictions of antigen-specific selection pressures. Third, we provide a policy-aware molecular surveillance framework to inform vaccine-strain selection and preparedness planning as interventions evolve (Timmis et al., 2017).

In summary, the pandemic provides a unique vantage point to understand how macro-level interventions influence viral evolution. By leveraging national policy differences, our work links transmission ecology to evolutionary outcomes in IBV-V and underscores the need to integrate genetic surveillance with policy metrics to anticipate and mitigate future antigenic drift and vaccine mismatch.

## Materials and methods

### Viral sequence data curation

Hemagglutinin (HA), neuraminidase (NA), and nucleoprotein (NP) gene sequences of the influenza B virus Victoria lineage (IBV-V), isolated from human hosts in China, the United States, and Australia between 2016 and 2024, were retrieved from the GISAID EpiFlu™ database (see Supplementary Table 1 for accession IDs). To mitigate temporal and geographical sampling bias and to enable a balanced comparative analysis, we implemented a stratified random sampling strategy. We applied a stratified sampling scheme with an upper cap of 100 sequences per country-year to limit over-representation in years with dense submissions. For country-years with fewer than 100 eligible sequences after quality control and de-duplication, all available sequences were retained (no up-sampling or imputation). Consequently, some strata (e.g., Australia-2020) have smaller sample sizes reflecting limited database availability. To ensure the comparability of cross-gene analyses, a subset of sequences with the three gene segments HA, NA, and NP was used when necessary. The exact number of sequences for each country, each year, and each gene is shown in Supplementary Table 2.

### Sequence alignment and genetic distance calculation

Multiple sequence alignment for each gene (HA, NA, NP) and each country dataset was performed independently using the MUSCLE algorithm as implemented in MEGA X software. All alignments were manually inspected and refined to ensure accuracy.

Pairwise genetic distances between all sequences within each aligned dataset were calculated using the Kimura 2-parameter (K2P) model in MEGA X’s Compute Pairwise Distances module. The treatment of gaps and missing data was set to pairwise deletion to maximize the use of available sequence information. The resulting pairwise distance matrices served as the foundation for all subsequent analyses of genetic divergence. For each country–gene–year subset, we computed all pairwise K2P distances and summarized them at the sequence level as the mean distance from each sequence to all other sequences sampled in the same calendar year.

### Multiple sequence alignment, phylogenetic tree construction, and visualization

The NA, HA, and NP gene sequences from China, the United States, and Australia were aligned using MUSCLE (v3.8.1551) with a balance between computational efficiency achieved by limiting the number of iterations (Federici et al., 2018) and enabling diagonal optimization. Subsequently, the maximum likelihood phylogenetic tree was constructed using IQ-TREE (v2.2.2.7), where the optimal nucleotide substitution model was automatically selected by Model Finder. Branch reliability was assessed through Ultra-fast Bootstrap with 1,000 resampling. In the visualization phase, epidemiological metadata were mapped onto a circular layout using the R-based ggtree package. Terminal and internal nodes were annotated with inferred sampling periods and colored, while a radial gradient fan was constructed to enhance visual differentiation among different branches. A geographic distribution heatmap was additionally overlaid on the outer ring.

### Analysis of temporal trends in genetic divergence

To quantify the temporal trend in genetic divergence, we performed a linear regression analysis using per-sequence genetic distance values. For each sequence, we calculated its mean pairwise genetic distance to all other sequences collected in the same calendar year. These sequence-level distance values were then regressed against the collection year (with 2016 as the baseline, year 0). The slope of the resulting regression line represents the trend of genetic distance change over time, which we use as a metric to compare the tempo of genetic divergence across different time periods and countries (Figure 3).

### Statistical comparison of genetic distances across epidemiological periods

To statistically evaluate the impact of pandemic-era interventions, sequences were categorized into three predefined epidemiological periods: Pre-pandemic (2016–2019), Pandemic (2020–2021), and Post-relaxation (2022–2024). This category captures the period of the strongest global non-pharmaceutical interventions (2020–2021) and regards 2022 as the beginning of the post-intervention transition period. The mean genetic distance was calculated for each period. The statistical comparisons (ANOVA and *post hoc* Tukey HSD test) were then performed using these sequence-level genetic distance values as the observational units. A one-way analysis of variance (ANOVA) was used to assess the overall significance of differences in mean genetic distance among these three periods. For datasets where the ANOVA indicated a significant overall effect (*p* < 0.05), we conducted *post hoc* pairwise comparisons between periods using Tukey’s Honest Significant Difference (HSD) test to control for family-wise error rates. The results of these pairwise comparisons, including *p*-values and 95% confidence intervals, are summarized in Figure 4. Although the distance values at the sequence level are not completely independent, using them as units for period-level comparisons provides a comparative assessment for evaluating differences between periods. All statistical tests were two-tailed, with a significance level of α = 0.05.

## Results

To assess the impact of COVID-19 non-pharmaceutical interventions (NPIs) on IBV-Victoria evolution, we stratified the study timeline (2016–2024) into three distinct epidemiological periods: Pre-pandemic (2016–2019), Pandemic/NPI (2020–2021), and Post-relaxation (2022–2024). All subsequent analyses and visualizations are framed within this temporal framework.

### Phylogenetic analysis reveals period-associated structuring of IBV-Victoria HA gene

**FIGURE 1:**
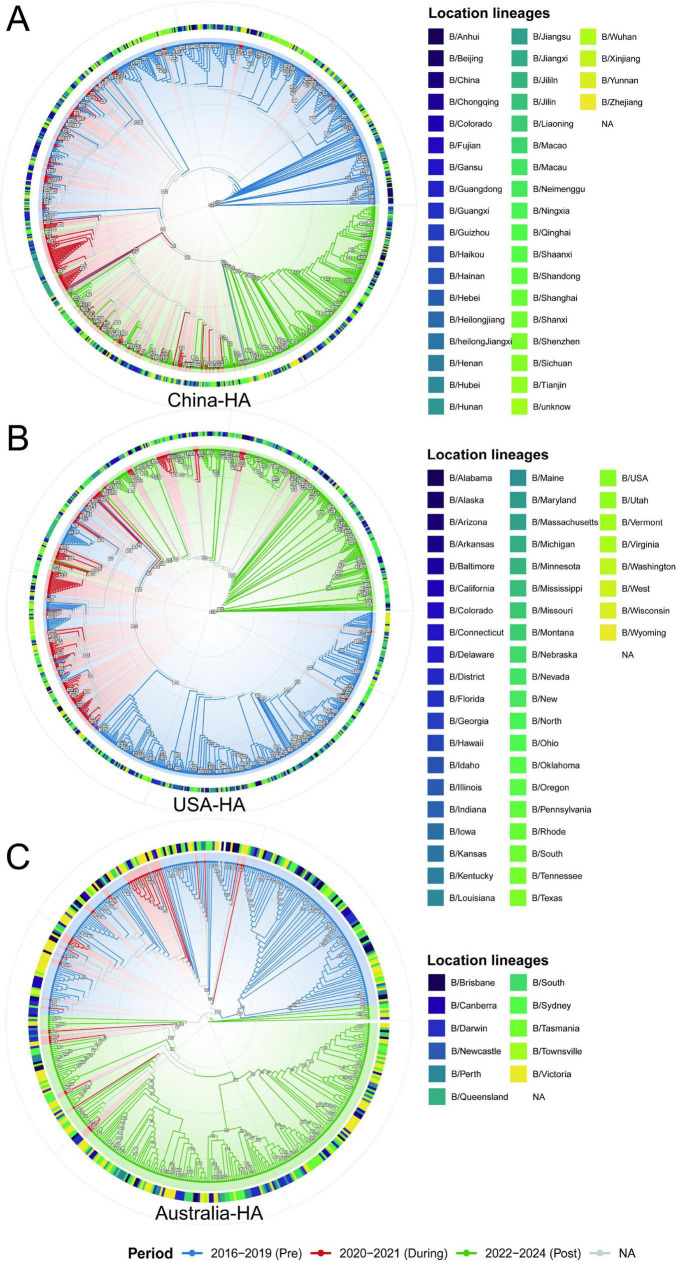
Phylogenetic structure of IBV-Victoria across periods and countries **(A–C)** hemagglutinin (HA). Country-specific circular maximum-likelihood trees with tips colored by period: pre-pandemic (2016–2019, blue), pandemic/NPI (2020–2021, red), and post-relaxation (2022–2024, green). Branch length denotes substitutions per site (subs/site). Panel titles indicate gene—country.

Country-specific maximum-likelihood phylogenies for the HA, NA, and NP genes reveal distinct temporal patterns. The HA gene trees (Figures 1A–C) exhibit period-associated clustering tendencies. In China and the United States, sequences from the post-relaxation period (2022–2024, green) show a tendency to form relatively separate branches, distinct from clusters comprised of pre-pandemic (2016–2019, blue) sequences. A similar pattern is observed in Australia, where post-relaxation sequences tend to cluster separately from those circulating during the stringent NPI period (2020–2021, red). In contrast, the NP and NA phylogenies (Supplementary Figures 1D–I) across all three countries exhibit extensive temporal intermixing, with sequences from all three periods scattered throughout the trees without forming period-specific clusters. Collectively, this visual pattern of period-associated clustering in HA-but not in NA or NP-provides a phylogenetic context for the gene-specific evolutionary dynamics quantified in the following analyses. Branch lengths represent substitutions per site.

### Year-wise genetic divergence of IBV-Victoria by gene

**FIGURE 2:**
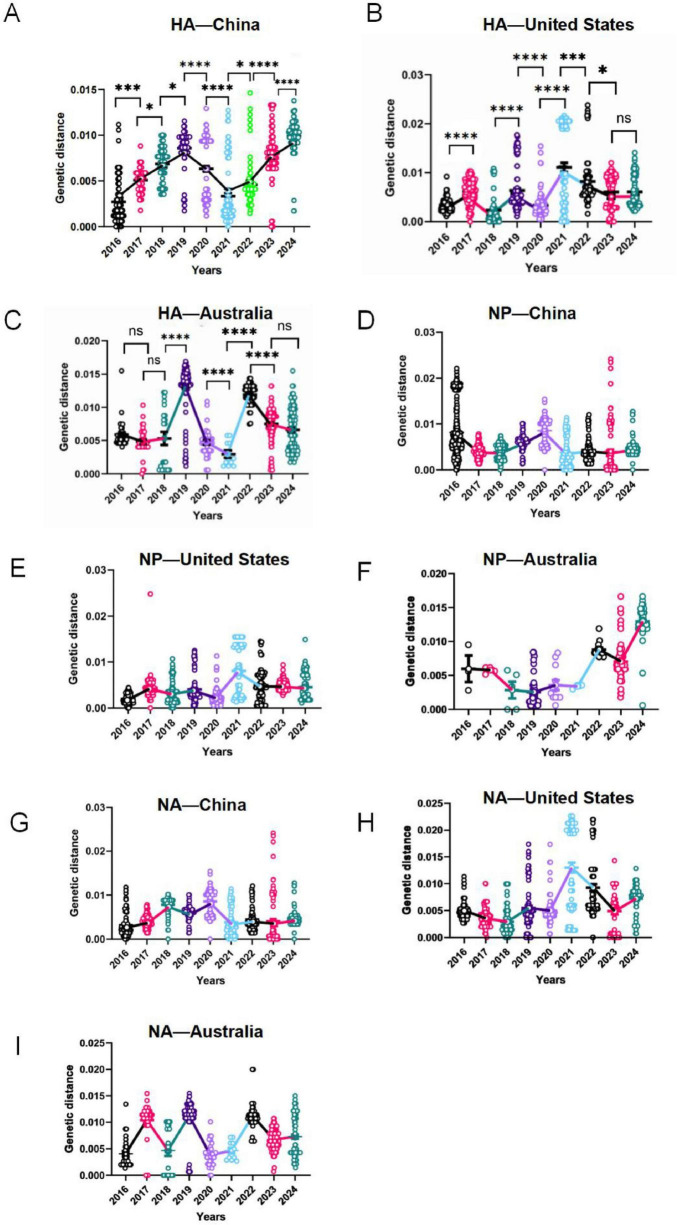
Year-wise genetic divergence of IBV-Victoria HA, NP, and NA in China, the United States, and Australia (2016–2024). **(A–C)** (HA); **(D–F)** Nucleoprotein (NP); **(G–I)** Neuraminidase (NA). Country-specific panels display year-wise distributions of genetic distance (substitutions per site, subs/site) from 2016 to 2024. The x-axis shows calendar year; the y-axis shows genetic distance. The vertical axis represents the average genetic distance of each sequence relative to all other sequences. Each dot represents one sequence, and each calendar year is rendered in a distinct color. *P < 0.05, ***P < 0.001, ****P < 0.0001.

Figure 2 shows year-wise distributions of per-sequence genetic distance (subs/site) from 2016 to 2024 for the Victoria lineage, stratified by gene in country-specific panels (HA: Figures 2A–C; NP: Figures 2D–F; NA: Figures 2G–I). Each dot represents one sequence; each calendar year is encoded with a distinct color. Across panels, HA divergence was relatively elevated around 2019, lower during 2020–2021, and increased again from 2022 to 2024 (Figures 2A–C). The annotations in Figure 2A mark tested contrasts between specific years and are concordant with the lower values in 2020–2021 followed by higher values after 2022. NP exhibits lower-amplitude interannual variability than HA (Figures 2D–F). Yearly distributions remain concentrated within a relatively narrow divergence band, with no persistent displacement centered on any particular year. NA shows modest, panel-specific fluctuations across years (Figures 2G–I). While local maxima occur in some years, there is no cross-panel pattern comparable to the HA pattern of change. Collectively, Figure 2 visualizes a pattern of higher HA genetic distance around 2019, lower during 2020/2021, and higher from 2022 onward. In contrast, NA and NP show comparatively smaller and less consistent year-to-year fluctuations.

### Temporal trend in genetic divergence by gene and country

**FIGURE 3:**
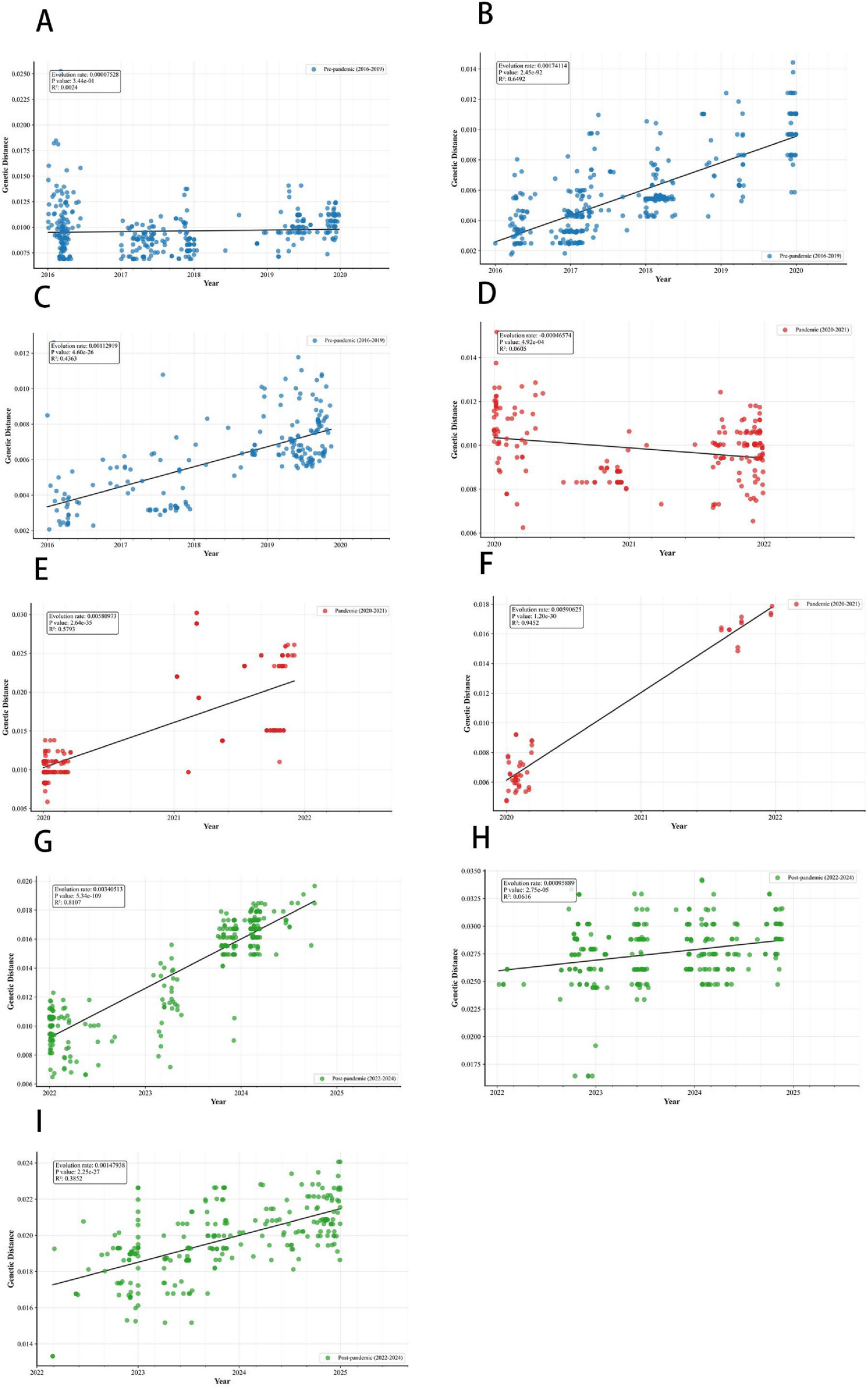
Period-stratified linear regressions of genetic divergence for IBV-Victoria hemagglutinin (HA), 2016–2024. **(A)** Pre-pandemic (2016–2019), China. **(B)** Pre-pandemic, United States. **(C)** Pre-pandemic, Australia. **(D)** Pandemic/NPI (2020–2021), China. **(E)** Pandemic/NPI, United States. **(F)** Pandemic/NPI, Australia. **(G)** Post-relaxation (2022–2024), China. **(H)** Post-relaxation, United States. **(I)** Post-relaxation, Australia.

Figure 3 arranges IBV-Victoria HA genetic distance (subs/site) against collection year to enable within-period, cross-country comparisons (rows = pre-pandemic 2016–2019, pandemic/NPI 2020–2021, post-relaxation 2022–2024; columns, left→right = China, United States, Australia). In the pre-pandemic subset (Figures 3A–C), all three countries show positive or weakly positive trends. During the pandemic/NPI period (Figures 3D–F), these trends are attenuated or heterogeneous across countries, and in several panels appear near flat. In the post-relaxation period (Figures 3G–I), positive trends re-emerge in each country. Given that 2022 marked the starting point for countries to transition from strict interventions to opening up, the trends during this period (2022–2024) reflect the early stage of the recovery of transmission dynamics. The re-emergence of positive slopes in the post-relaxation period across all three countries indicates a recovery of HA genetic divergence following the lifting of NPIs.

**FIGURE 4:**
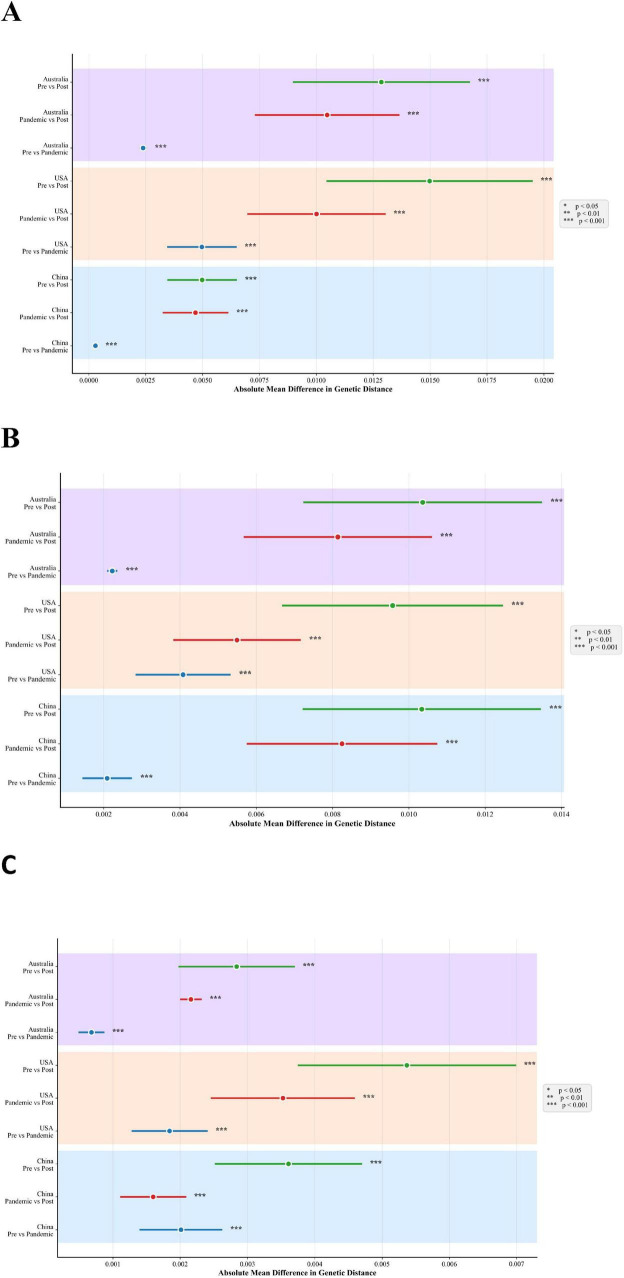
Period contrasts in mean genetic distance for IBV-Victoria by gene and country (2016–2024). **(A)** Hemagglutinin (HA). **(B)** Neuraminidase (NA). **(C)** Nucleoprotein (NP). For each country—China (blue), United States (orange), Australia (purple)—we show three pairwise contrasts between epidemiological periods: Pre (2016–2019) vs. NPI (2020–2021), Post (2022–2024) vs. NPI, and Post vs. Pre. Points represent the absolute mean difference in genetic distance (substitutions/site); horizontal lines denote 95% confidence intervals. Significance thresholds are indicated by asterisks (***p < 0.001).

Figure 4 presents pairwise contrasts in absolute mean genetic distance between epidemiological periods (Pre, NPI, Post) for HA, NA, and NP genes across China, the United States, and Australia. For HA (Figure 4A), multiple period comparisons—particularly Post vs. Pre and Post vs. NPI—show statistically significant differences (marked by asterisks) in all three countries. This indicates that HA genetic distances shifted substantially between periods, reflecting altered evolutionary dynamics associated with changes in public health measures. In China and Australia, where strict COVID-19 controls were initially implemented and later relaxed, HA rate of genetic distance change decreased during the NPI period and then increased post-2022, though the rebound patterns differed-gradual in China and abrupt in Australia following localized outbreaks. In contrast, the United States adopted more heterogeneous and inconsistently strict measures, with its policies relaxing rapidly after 2021. As a result, its patterns in 2022 and beyond reflected an early and uneven recovery dynamic of transmission, leading to greater fluctuations in the rate of change of HA genetic distance throughout the study period. In comparison, NA (Figure 4B) and NP (Figure 4C) show consistently smaller and often non-significant genetic distance differences between periods. Most confidence intervals are narrow and centered near zero, suggesting that the temporal shifts in genetic distance for these genes were subtle and not consistently aligned with policy changes, in stark contrast to the pronounced period-associated shifts observed in HA. These period contrasts in HA are concordant with the period-specific trends in Figure 3 (attenuated during 2020–2021 and re-increasing in 2022–2024), while NA/NP show smaller effects.

## Discussion

The COVID-19 pandemic and associated non-pharmaceutical interventions (NPIs) created a global natural experiment for respiratory virus evolution. Our analysis of IBV-Victoria sequences from 2016 to 2024 across three countries reveals a gene-specific modulation of evolutionary dynamics. HA genetic divergence showed a pattern of deceleration during the peak intervention period (2020–2021) followed by an increase thereafter, whereas NA and NP exhibited comparatively smaller and less consistent changes. Pairwise between-period contrasts further indicate that HA exhibits the largest Pre-NPI and Post-NPI differences within countries, with NA/NP differences modest.

These observations are consistent with population-genetic expectations. The sharp decline in HA divergence is consistent with a scenario of reduced effective population size under stringent NPIs, which attenuates positive selection and amplifies genetic drift. As the primary antigenic target, HA is under intense immune pressure during normal transmission; NPIs may have relaxed this pressure through the suppression of transmission chains. The subsequent increase post-2022 coincides with the restoration of transmission opportunities and immune-driven selection. The stark contrast with the much more subdued temporal profiles of NA and NP-genes subject to stronger structural constraints and slower antigenic drift-underscores that the pandemic’s impact was not a genome-wide effect, but a targeted modulation of the most dynamically evolving antigenic component.

While the direction of HA’s response-downturn followed by rebound-was consistent across China, the United States, and Australia, the magnitude and timing of these shifts offer nuanced insights. The pronounced and synchronized “V-shaped” trajectory in China and Australia is broadly consistent with the implementation of more uniform and stringent NPIs, which created strong, population-wide transmission suppression. In contrast, the earlier and more volatile pattern in the United States is consistent with its decentralized and heterogeneous policy landscape, resulting in a “patchwork” of transmission dynamics that afforded the virus more opportunities to evolve. Although our ecological design precludes definitive causal attribution, these cross-country patterns are consistent with the hypothesis that the intensity and consistency of transmission suppression, rather than the mere presence of interventions may be important factors associated with the tempo of viral antigenic evolution.

Our period-based analysis simplifies complex national timelines. The inclusion of 2022 in the post-relaxation phase captures early recovery while acknowledging its transitional nature. Other factors, such as pandemic-related shifts in influenza surveillance intensity or post-pandemic immunity landscapes, could have contributed to the observed genetic patterns. While our three-gene framework helps isolate antigen-specific signals, future work integrating epidemiological and mobility data is needed to separate these concurrent drivers.

Our findings have practical implications for public health. First, policy-aware genomic surveillance-interpreting HA divergence in the context of intervention timing-may improve the anticipation of antigenic drift during future public-health transitions. Second, the post-2022 rebound in HA underscores a potential increase in antigenic-drift and vaccine-mismatch risk during reopening, arguing for enhanced monitoring and timely candidate vaccine virus evaluation. Third, routine gene-resolved analytics that benchmark HA against NA/NP can help separate antigen-specific selection from background genomic drift, refining early-warning signals.

This study has several limitations. Despite stratified sampling, heterogeneity in sequence submissions may introduce bias. We note that some country-year strata contain fewer sequences due to limited availability in GISAID (e.g., Australia-2020), which may increase sampling variability; therefore, year-specific estimates from sparse strata should be interpreted cautiously, with emphasis on multi-year and period-level patterns. Our use of linear regression on per-sequence genetic distance values provides a descriptive metric of temporal trend but differs from model-based substitution-rate estimates. Furthermore, we did not jointly analyze incidence, NPI stringency, or mobility data; thus, policy-evolution links remain associative. Additionally, the per-sequence genetic distance values used in our statistical comparisons are not fully independent, as individual sequences contribute to multiple pairwise calculations. This non-independence may affect the precision of *p*-values. However, our primary conclusions—the distinct response pattern of HA compared to NA/NP, and its consistency across countries—are based on large and consistent effect sizes, which remain robust to this limitation. Moreover, our definition of epidemiological periods, while aligned with major policy transitions, represents a simplification of complex, country-specific timelines. Finally, gene-level analyses cannot localize selection to specific epitopes, warranting future site-level studies for mechanistic insight.

In conclusion, by comparing three countries with divergent intervention histories, we provide evidence consistent with the idea that macro-level transmission shifts during the COVID-19 era may have left a detectable, gene-specific molecular signature on IBV-Victoria. These results underscore the value of integrated genetic-epidemiological surveillance to anticipate influenza evolution and strengthen preparedness for future epidemics.

## Data Availability

The original contributions presented in this study are included in this article/Supplementary material, further inquiries can be directed to the corresponding authors.

## Supplementary material

Supplementary Figure 1 Phylogenetic trees of influenza B/Victoria NA and NP genes across China, the United States, and Australia (2016–2024).

Supplementary Table 1 Complete list of influenza B/Victoria lineage sequences used in this study, by country and gene segment.

Supplementary Table 2 Summary of sequence counts for HA, NA, and NP genes, and the subset of isolates with complete three-gene data, by country and year.
