# Correction: Deceleration and rebound of hemagglutinin divergence in influenza B/Victoria across COVID-19 NPI phases (2016–2024)

**DOI:** 10.3389/fmicb.2026.1819187

**Published:** 2026-03-10

**Authors:** Frontiers Production Office

**Affiliations:** Frontiers Media SA, Lausanne, Switzerland

**Keywords:** apoptosis, caspase-3, infectious bursal disease virus (IBDV), innate immunity, IRF7, viral replication

The affiliation of reviewer Abdul Bari Hejran, was erroneously given as Al-Farabi Kazakh National University, Kazakhstan. The correct affiliation is Helmand University, Helmand, Afghanistan.

The original version of this article has been updated.

