# Supplementary figures and images for: Deceleration and rebound of hemagglutinin divergence in influenza B/Victoria across COVID-19 NPI phases (2016–2024)
Source: Front Microbiol. 2026 Feb 25;17:1791792. doi: 10.3389/fmicb.2026.1791792 (PMC13011507; doi:10.3389/fmicb.2026.1791792)

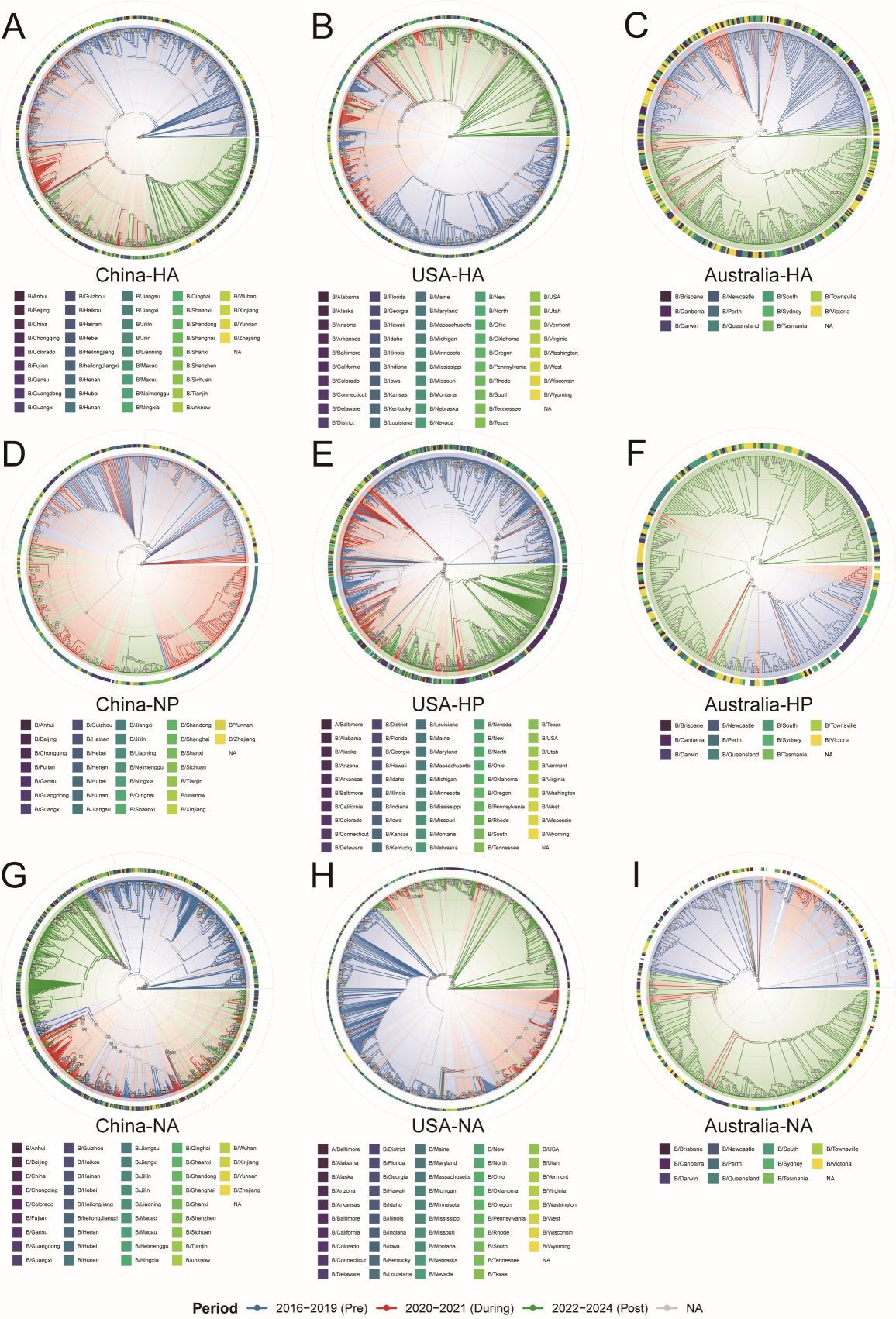

Supplement: Supplementary Figure 1 — Phylogenetic trees of influenza B/Victoria NA and NP genes across China, the United States, and Australia (2016–2024). [file Image_1.TIFF]
